# Characteristics of Mg-Zn-Ca-Pr Alloy Synthesized by Mechanical Alloying

**DOI:** 10.3390/ma17215336

**Published:** 2024-10-31

**Authors:** Sabina Lesz, Małgorzata Karolus, Bartłomiej Hrapkowicz, Tomasz Gaweł, Michał Bielejewski, Rafał Babilas, Tymon Warski, Julia Popis

**Affiliations:** 1 Department of Engineering Materials and Biomaterials, Silesian University of Technology, 18a Konarskiego Street, 44-100 Gliwice, Polandtomasz.gawel@us.edu.pl (T.G.); rafal.babilas@polsl.pl (R.B.); julia.popis@polsl.pl (J.P.); 2 Institute of Materials Science, University of Silesia, 75 Pułku Piechoty 1A Street, 41-500 Chorzów, Poland; 3 Institute of Molecular Physics, Polish Academy of Sciences, 17 Smoluchowskiego Street, 60-179 Poznań, Poland; bielejewski@ifmpan.poznan.pl; 4 Łukasiewicz Research Network, Institute of Non-Ferrous Metals (Ł-IMN), 5 Generała Józefa Sowińskiego Street, 44-121 Gliwice, Poland; tymon.warski@imn.lukasiewicz.gov.pl

**Keywords:** Mg-based alloys, mechanical alloying, phase analysis, DSC

## Abstract

Magnesium-based materials are an interesting solution in terms of medical applications. Alloys that are hard to obtain via standard means may be manufactured via mechanical alloying (MA), which allows the production of materials with complex a chemical composition and non-equilibrium structures. This work aimed to investigate materials obtained by the MA process for 5, 8, 13, and 20 h in terms of their phase composition and changes during heating. The results of thermal XRD analysis were in the temperature range between 25 and 360 °C, which revealed MgZn_2_, PrZn_11_, Ca_2_Mg_5_Zn_13_, and Ca phases as well as α-Mg and α-Zn solid solution. The structural analysis features the powder morphology of the analyzed samples, showing cold-welding and fracturing processes leading to their homogenization, which is supported by the EDS results. The base Mg-Zn-Ca alloy was modified by different additions, but a thorough analysis of the influence of praseodymium on its thermal properties has not yet been performed. We chose to focus on Pr addition because it belongs to low-toxicity rare earth metals, which is an essential feature of biomaterials. Also, the Ca_2_Mg_5_Zn_13_ phase is not fully known, as there are no crystallographic data (hkl). Therefore, the investigation is important and scientifically justified.

## 1. Introduction

Magnesium is a crucial material for the human body and its metabolic processes, and as such, it seems like a very interesting solution as a biodegradable material. Not only is it lightweight, but it also has good mechanical properties comparable to human bones, and its corrosion rate can be controlled [1,2,3,4]. An effective strategy for improving the corrosion resistance of Mg-based alloys, which will contribute to reducing the amount of gaseous hydrogen, is to modify the chemical composition by introducing alloying additives to pure magnesium [5,6,7,8,9,10].

The material for a biomedical implant should be properly tested before its actual use. It is necessary to investigate the relationship among chemical composition, microstructure, corrosion, and mechanical properties. The key problem with metal implants is their corrosion, the occurrence of which is related to the unfavorable nature of the human body. Introduced implants are exposed to conditions comparable to a salty marine environment. In body fluids, there are, e.g., chloride, sodium, potassium, calcium and magnesium ions, and phosphates. Relatively high body temperature also has an unfavorable effect [1,2,11,12]. Implants are exposed to the following damage: pitting, fretting, galvanic corrosion, and fatigue [13,14,15,16,17].

To choose the right composition and alloying additives of the alloy for the implant, it is important to determine its type, working environment, and the desired properties. The alloying process also has a significant impact on the properties of the implant. Not all methods can produce an alloy with the required criteria. An example of an alloy usable in the human body is Mg-Zn-Ca [18,19,20]. Zinc is biocompatible; additionally, the Mg-Zn alloys harden in the aging process. Moreover, Zn addition strengthens the overall alloy as well as casting capacity. It can cause cracking, however, when it exceeds a certain threshold. Another element typically associated with magnesium alloys is calcium, as it has a beneficial effect on the alloy microstructure. When added to the Mg-Zn alloys, it also increases its creep resistance and hardness. In ternary alloys, the solubility of zinc in magnesium increases along with calcium concentration. Both Zn and Ca can positively affect the healing process, bone mending, as well and overall stability under physiological conditions [2,21,22].

Among the many methods capable of producing materials with a non-equilibrium structure is mechanical alloying (MA). This technique makes it possible to create a microstructure or even a nanostructure. The basis of the method is cyclical deformations (welding, crushing, and re-welding); these processes result in the reduction in grain sizes and the creation of new grain boundaries. Precisely composed and pure materials can be achieved through MA [23,24,25].

The properties of materials produced through mechanical alloying can be defined during the process design. It is very important for implantology, where the selection of appropriate, specific features of the material for the treated patient not only allows lessened costs of treatment but also reduces or eliminates complications. Implants often have a complex shape that is difficult to manufacture. The process of mechanical alloying in combination with consolidation operations, i.e., SPS sintering, enables the production of near-net-shaped elements. The implants are usually made from three main material classes, namely metal, ceramic, and polymers. For orthopedic and dental applications, metal implants are mainly used because of their high mechanical and strength properties, e.g., stainless steels, Cr-Co alloys, and titanium alloys. However, their major drawback is their lack of biodegradability, which may force the patient to undergo another surgery to improve or remove the implant. This may cause undesirable and negative reactions, thus contributing to the treatment time extension and cost increase. In the worst case, serious complications can occur. At present, considerable research is directed toward the development and production of biodegradable implants with low density and high bioactivity that also retain their mechanical properties [26,27,28,29,30,31,32].

Mechanical alloying enables a very high rate of phase transformations associated with a low rate of diffusion in metals at a temperature of several dozen degrees Celsius. The process is facilitated by a significant contact area between processed powders, which further increases during the thinning of the plate structures present. Increased dislocation density and higher-than-equilibrium vacancies lead to a rise in the diffusion rate. Another beneficial feature is the local temperature increase caused by the friction and plastic deformation of the powders. The size of the resulting grains also depends on the type of mill in which they were produced. Each of them has its specific milling energy. Depending on the milling intensity, the grain size and deformation can be different. The greater the milling intensity, the smaller the grain, and the greater the deformation. The mass of the ground powder also plays a significant role. A greater amount of powder, which behaves like a viscoelastic layer, reduces the impact force, which increases along the milling frequency [23,24,25].

Various additives can be added to the magnesium-based alloys, severely affecting their properties, e.g., Ni, Cr, Co, Be, Al, and Y. For example, yttrium has a relatively high solubility in magnesium, enhancing its strength, mostly due to solid-solution hardening [33,34]; however, it may cause a reduction in the cohesive strength of the grain boundaries when the content exceeds a certain threshold. Among the mentioned additives, the rare earth metals (REE) are very interesting, such as Gd, Nd, and others. Gadolinium may be used as a substitute for Y in Mg-based alloys, as it is not prone to forming oxides during the alloy processing, and it exhibits strengthening properties like Y [35,36,37,38,39]. Neodymium, on the other hand, forms stable precipitates in Mg with its decreased solubility. Moreover, its phases are thermally stable and may decrease galvanic corrosion in certain alloys [34,35,40,41]. Praseodymium is another REE used mainly to produce high-strength magnesium alloys. The studies performed by Ahmad revealed that even 1 at. % Pr addition causes hardness to increase due to the grain refinement [42]. Other research stated that Pr improves overall mechanical performance and stability in high temperatures [42]. As such, praseodymium may be an interesting alloying addition to magnesium-based alloys for medical applications. The only concern that is raised with the REEs is their low to mild toxicity. Pr, however, has no known biological role and has low toxicity as compared to other REEs. Nakamura conducted studies on Pr toxicity in 1997, stating that Pr may accumulate in organs [43]. Studies on rats showed that the food allowance for praseodymium is 600 mg kg^−1^. However, it is important to note that the studied rats were exposed to considerable amounts of Pr in a short time. The experimental exposure limit for praseodymium is MG63 > 1000 LD50/μM. Overdose of praseodymium may provoke pulmonary embolisms, especially during long-term exposure. It can be a threat to the liver as well when it accumulates in the body [5].

Research shows that cerium, praseodymium, and yttrium may cause severe hepatotoxicity [44], and with the advancement of research techniques, more and more elements may be toxic. However, alloys containing Ce, Pr, or Y can also be safe if their release from the alloy is within the tolerance limit. The total amount of these rare earth elements (Ce, La, Nd, Pr, and Y) should not exceed the value of 4.2 mg per day [6,41].

This paper is part of a preliminary study concerning quaternary Mg-based alloys with a nominal composition of Mg_65_Zn_30_Ca_4_Pr_1_. This study aimed at elucidating the processes and phase changes that occur during the heating of the Mg_65_Zn_30_Ca_4_Pr_1_ alloy when prepared by high-energy mechanical alloying (HEMA). Additionally, the goal was to assess its potential suitability as a sintering material, ultimately to create a potential candidate for biomaterial applications.

## 2. Materials and Methods

The alloy powder with a composition of 65% Mg, 30% Zn, 4% Ca, and 1% Pr (in at.%) was fabricated by the high-energy mechanical alloying (HEMA) method at room temperature (RT) using the 8000D Ball Mill (SPEX SamplePrep, Metuchen, NJ, USA). Commercially available powders of Mg (Alfa Aesar, −20 + 100 mesh, 99.8%), Zn (Alfa Aesar, −100 mesh, 99.9%), Ca (Chempur, irregular pieces < 1 cm, 99%), and Pr (Chemat, −200 mesh, 99.9%) were utilized as the starting materials. The appropriate amounts of elemental powders with a total mass of about 10 g were weighed, mixed, and poured into a round-bottom stainless-steel container, with grinding balls made of 316L stainless steel and with a 10 mm diameter, in an argon atmosphere. The ball-to-powder ratio was established as 10:1. The powders were subjected to milling in various cycles of 5, 8, 13, and 20 h. The cycle consisted of 1 h milling and 30 min intervals of cooldown breaks.

The X-ray diffraction (XRD) analysis was performed using the Empyrean diffractometer (PANalytical, Almelo, The Netherlands). Co-K α (Co-K α; λ = 1.79 Å; voltage 40 kV; anode current 30 mA; angle step of 0.05°; time 100 s) and Cu-K α (λ = 1.54056 Å; voltage 45 kV; anode current 40 mA; the angle step of 0.02°; time per step, 1000 s/step) radiation was used for elemental powders and synthesized Mg_65_Zn_30_Ca_4_Pr_1_ alloy powders, respectively. A PIXCell counter with the step-scanning method in the 15 to 100 ° 2θ angle range was applied. The measurements were performed using Bragg–Brentano geometry.

The phase analysis of initial powders and milling products was performed with the High Score Plus PANalytical software (version 4.0, PANalytical, Almelo, The Netherlands) and the ICDD PDF5+ 2024 database (International Centre for Diffraction Data, Newtown Square, PA, USA). The temperature analysis was performed using the same diffractometer as the Anton Paar HTK 450 thermal camera in the 25–360 °C temperature range. The measuring range included the location of measurements performed in a range of the characteristic diffraction lines of the identified phases’ presence, i.e., 2θ angles from 15 to 80°.

The thermal analysis of the Mg_65_Zn_30_Ca_4_Pr_1_ alloy powder milled for 5, 8, 13, and 20 h was performed by differential scanning calorimetry (DSC) method using NETSCH Jupiter STA 449 F3 (Netsch, Selb, Germany) at a heating rate of 10 °C/min up to 450 °C under a purified argon atmosphere. The crystallization process was examined by applying the isochronal DSC (NETZSCH STA 449F3) method with heating rates of 10, 20, and 40 °C/min. The activation energies (Ea) of the crystallization process of the Mg_65_Zn_30_Ca_4_Pr_1_ alloy powder after milling for 5 h were obtained using the Kissinger method [42,45]. The samples were heated from 150 to 450 °C, using different rates of 10, 20, and 40 K/min. For this purpose, the exothermal reaction was plotted according to the following formula [43]:

(1) $$ln\left(\frac{\beta }{T_{p}^{2}}\right)=ln\left(\frac{A_{0}R}{E_{a}}\right)-\frac{E_{a}}{\left(RT_{p}\right)}$$

Equation (1) is known as the Kissinger model and allows us to estimate the activation energy of the crystallization process, where β—heating rate, $T_{p}$—temperature of the crystallization peak, $E_{a}$—activation energy, *R*—gas constant, and $A_{0}$—pre-exponential factor. By linear fitting of $ln\left(\beta /T_{p}^{2}\right)$ vs. $1/T_{p}$ curves, the average activation energy *E_a_* of the process was determined from the slopes of these curves.

The morphology of elemental powders and milling products was examined using the scanning electron microscope (SEM) SUPRA 35 (Carl Zeiss, Oberkochen, Germany), equipped with UltraDry EDS Detector (Thermo Scientific™ EDX UltraDry, Waltham, MA, USA). Five EDS scans were performed in each sample to check the homogeneity. Representative images of selected scribe areas were analyzed and are shown. The measurement uncertainties for the main and major elements in wt.% are 2% and 4%, respectively, and for minor and trace elements, they are in the ranges of 10–20% and 50–100%, respectively. Observations of morphology alloy powders prepared by MA were performed in a Zeiss Supra 35 scanning electron microscope (SEM) equipped with an EDS diffuse X-ray detector. Maps of element concentration distributions of the Mg-based alloy were studied with EDS analysis (SEM).

The particle size distribution of powders of the Mg_65_Zn_30_Ca_4_Pr_1_ alloy produced by the MA method was determined by the laser diffraction method using a Laser Particle Sizer Analysette 22 MicroTec plus (Fritsch, Weimar, Germany), which has a measuring range of 0.8–2000 μm. The study used high-purity (99%) ethyl alcohol, and the samples were dispersed using ultrasound to minimize the risk of agglomeration. One test cycle consisted of four measurements using both red and green lasers in coarse and fine modes. For each sample, five measurement cycles were carried out using the Fraunhofer method. The obtained results were recalculated with MaScontrol software (Fritsch, Idar-Oberstein, Germany) to generate the particle-size distribution curves of the test samples.

## 3. Results and Discussion

**Figure 1:**
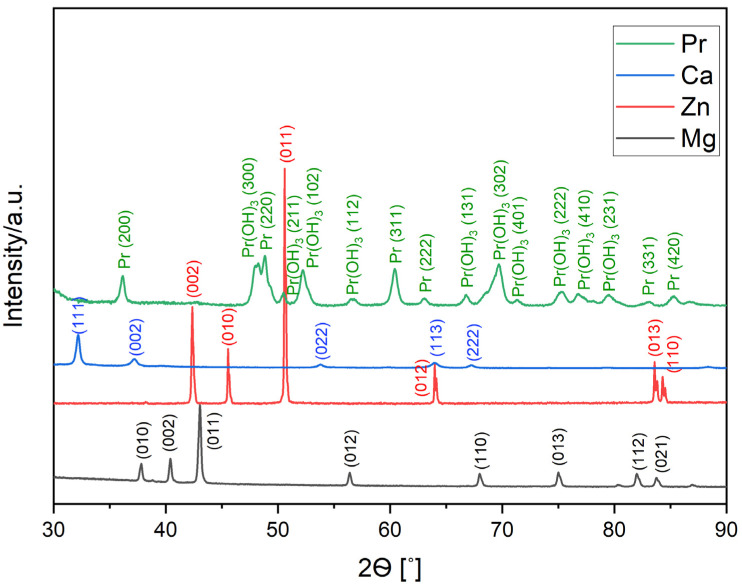
XRD patterns of the initial powders of Mg, Zn, and Pr and Ca pieces.

The XRD patterns of pure Mg (98-005-2260), Zn (98-005-2259), and Pr (00-002-0692) powder and Ca (98-042-6932) pieces are presented in Figure 1. Apart from peaks for the major elements (Mg, Zn, and Ca), peaks for Pr(OH)_3_ (01-083-2304) phase were also identified. Praseodymium oxidizes very rapidly, forming Pr(OH)_3_ hydroxide in a high-humidity atmosphere.

**Figure 2:**
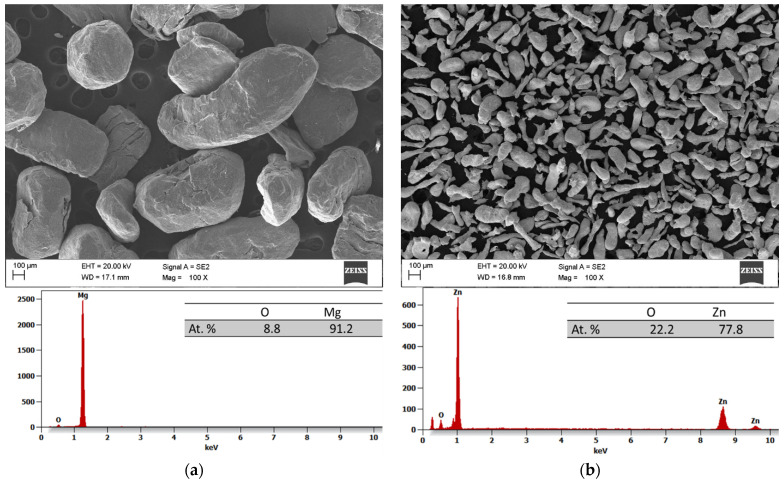

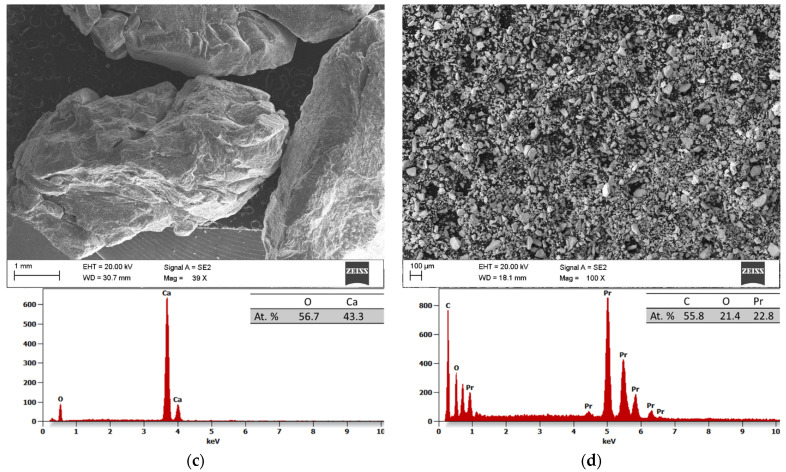
Scanning electron microscopy (SEM) and energy dispersive spectrum (EDS) analysis of the metallic elements used to fabricate the alloy: initial powders of Mg (**a**), Zn (**b**), Ca (**c**), and Pr (**d**).

The results of the scanning electron microscopy (SEM) and the energy dispersive spectroscopy (EDS) analyses of the initial powders before the process of MA are depicted in Figure 2a–d. All the powders have irregular shapes, with magnesium, zinc, and praseodymium powder particles measuring about 950 ± 420 (Figure 2a), 168 ± 124 (Figure 2b), and 23 ± 18 (Figure 2d) μm, respectively. Meanwhile, the pieces of calcium presented an irregular shape and a size from 6500 to 10,000 μm (Figure 2c).

**Figure 3:**
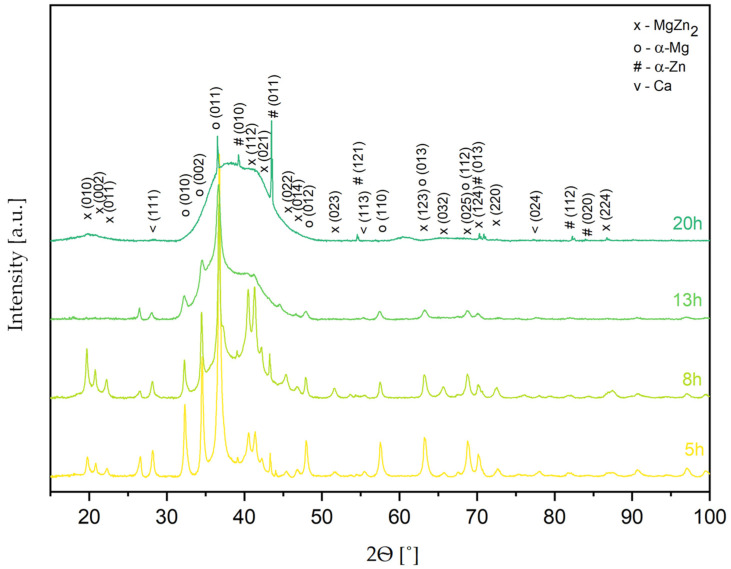
The XRD patterns of the Mg_65_Zn_30_Ca_4_Pr_1_ alloys were obtained by MA for different milling times (5, 8, 13, and 20 h).

Figure 3 shows the XRD patterns of the Mg_65_Zn_30_Ca_4_Pr_1_ alloy powder obtained by MA for different milling times (5, 8, 13, and 20 h). Based on the XRD analysis results, it was found that the structure of alloy powder is multiphase (Figure 3), in which the following phases were identified: α-Mg solution solid (P63/mmc), α-Zn solution solid (P63/mmc), MgZn_2_ (P63/mmc) phase, and traces of unreacted Ca (Fm3m). After 5 h of milling, a broadening of the peaks and a decrease in the intensity of the Mg peaks was observed, and this process continued until 20 h of milling. The observed maximum broadening between 30 and 50° (2θ), characteristic of amorphous phase presence, was the most visible for the powder alloy milled for 20 h. Therefore, the Mg_65_Zn_30_Ca_4_Pr_1_ alloy milled for 20 h was selected as a representative thermal analysis sample to observe possible phase changes occurring during the processes.

The broadening of the peaks and the decrease in their intensity are the results of the mechanical deformation of the powder during milling, leading to the refinement of the crystallite size and the increases in microstrain (accumulation of internal strain). The formation of a nanocrystalline solid solution can also lead to a broadening of the peaks. The deformation associated with mechanical alloying generates structure defects at the atomic scale. They induce energy and lead to extended solid solubility and therefore the supersaturation of solid solutions. The possible methods of energy relaxation in severely deformed alloys are recrystallization or phase transformation. As reported in the literature, the formation of crystalline phases is induced by thermally prompted nucleation and growth, while the amorphous phases are formed at the low formation rates of their crystalline counterparts [45], and the powders obtained via mechanical alloying are in a metastable phase where the transition to the crystalline state may be triggered by supplying activation energy through heating, thus triggering diffusive processes [44]. In mechanical alloying, amorphous and crystalline alloys act as alternatives, as high-nucleation-rate phases are a very good way to relieve the system from the excess energy, while more complex phases with many elements are not viable; hence, an amorphous phase is formed.

**Figure 4:**
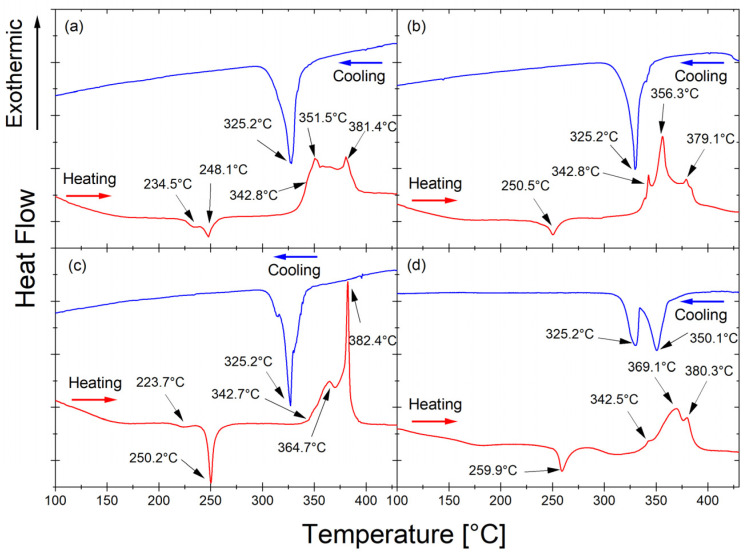
The DSC curves of the Mg_65_Zn_30_Ca_4_Pr_1_ alloy powder milled for 5 h (**a**), 8 h (**b**), 13 h (**c**), and 20 h (**d**) were recorded during heating (red line, right) and cooling (blue line).

The thermal properties of the Mg_65_Zn_30_Ca_4_Pr_1_ alloy powder milled for 5, 8, 13, and 20 h upon heating (red line, right) and cooling (blue line) were studied by DSC at a heating rate of 10 °C/min and are presented in Figure 4.

The exothermic peaks occurring at temperatures 234.5 °C (Figure 4a) and 223.7 °C (Figure 4c) are associated with the irreversible phase transformation. The exothermic peaks occurring after all milling times at 248.1 °C (Figure 4a), 250.5 °C (Figure 4b), 250.2 °C (Figure 4c), and 259.9 °C (Figure 4d) are related to the irreversible transformation from the amorphous phase to the crystalline MgZn_2_ phase. The endothermic peaks at 342.8 °C (Figure 4a), 342.8 °C (Figure 4b), 342.7 °C (Figure 4c), and 342.5 °C (Figure 4d) originate from the melting of the phase present in the alloy. The endothermic peaks at 351.5 °C (Figure 4a), 356.3 °C (Figure 4b), 364.7 °C (Figure 4c), and 369.1 °C (Figure 4d) occurred after all times milling as a result of the transformation of the reversible phase occurring in the alloy. The endothermic peaks at 381.4 °C (Figure 4a), 379.1 °C (Figure 4b), 382.4 °C (Figure 4c), and 380.3 °C (Figure 4d) most likely represent the melting of the non-equilibrium MgZn_2_ phase (Figure 3) [46]. During heating, the same processes occur at slightly different temperatures as the alloy milling time increases. The variations in transformation temperature can be attributed to the energy stored within the milled powder. During the milling process, the powder particles experience high-energy collisions when trapped between the milling media. This transferred energy causes work hardening and particle fracture. Moreover, milling introduces various crystal defects, such as dislocations, vacancies, and stacking faults, as well as new surfaces and grain boundaries [47]. It is difficult to discern glass transition temperature from the DSC curves due to the presence of crystalline phases in addition to the amorphous phase in the Mg_65_Zn_30_Ca_4_Pr_1_ alloy. The base alloy without the addition of praseodymium, with the composition Mg_66_Zn_30_Ca_4_, exhibits a somewhat different DSC characteristic [46].

The lack of a visible transformation at this temperature during cooling (blue curves in Figure 4) means that the transformation recorded during heating (red curves in Figure 4) around 250 °C is an irreversible transformation. The solidus temperatures of 325.2 °C (Figure 4a–c), 325.2 °C, and 350.1 °C (Figure 4d) that were determined from the cooling curves (blue line) are related to the solidification of the phase present in the Mg_65_Zn_30_Ca_4_Pr_1_ alloy. The cooling rate influences the shape and position of the exothermic peak. The exothermic nature of the transformation, the shape of the peak, and the fact that it is an irreversible process indicate that it is related to crystallization. The reversible transformation probably corresponds to the phase transition. The solidification peaks detected on cooling agree with the peaks that appeared during heating.

The DSC curve for the alloy after 13 h of milling (Figure 4c) indicates stronger and slightly different processes than for the other milling times. After milling for 13 h, the material began to amorphize significantly, so a minimum temperature of 250 °C may indicate a stronger effect of the formation of a new phase. With progressive amorphization of the material, homogenization of the material may occur, which may favor the formation of a new order/new phase. The amorphous structure of the alloy after 13 h of milling (Figure 4c) and after 20 h of milling (Figure 4d) is evidenced by a broad peak in the range of 30–47°, shown in Figure 3. The XRD diagram looks different for the remaining milling times (5 and 8 h). Only in the alloy that underwent 20 h of milling was there is a clear formation of an amorphous structure (Figure 3). However, the Mg_65_Zn_30_Ca_4_Pr_1_ alloy that underwent 20 h of milling time showed different DSC scans. The cooling peak was split into two at 325.2 °C and additionally 350.1 °C, which appeared in the alloy after 13 h of milling. The order resulting from the heating of an amorphous material was completely different from the order in heated materials in intermediate states (Figure 4a–c), and consequently, the cooling process took place differently, i.e., a different transformation.

**Figure 5:**
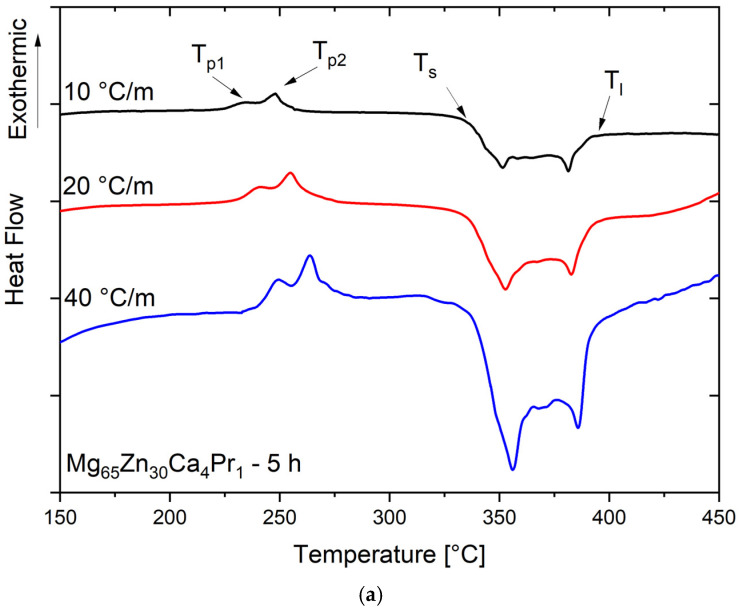

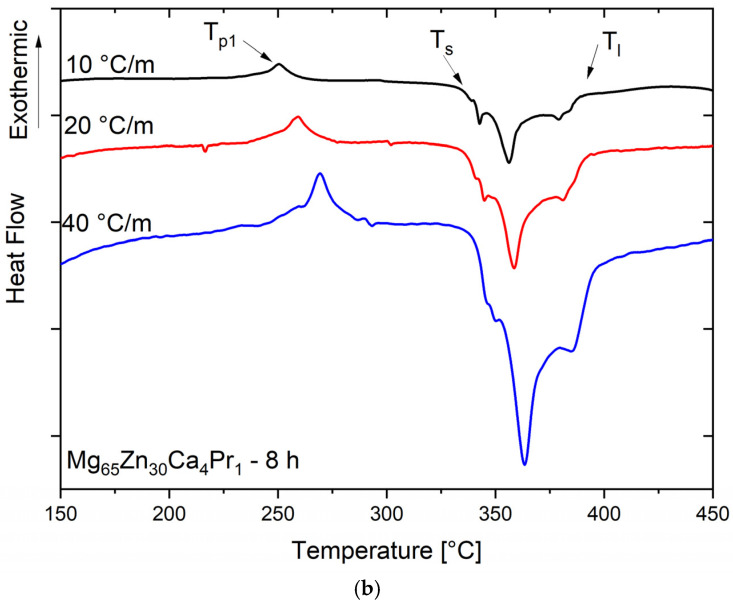
DSC curves for the Mg_65_Zn_30_Ca_4_Pr_1_ alloy powder milled for 5 h (**a**) and 8 h (**b**), measured with different heating rates: β = 10, 20, and 40 K/min.

The DSC scans with different heating rates β for the Mg_65_Zn_30_Ca_4_Pr_1_ alloy powder milled for 5 h and 8 h are presented in Figure 5a, and Figure 5b, respectively. The samples of the alloy powder milled for 5 h presented two distinct exothermal peaks (T_p1_ and T_p2_), and this behavior is independent of the applied heating rate, suggesting a crystallization process with two distinct exothermal reactions (Figure 5a). With the increase in heating rate β, the position of both peaks T_p1_ and T_p2_ was shifted to the higher temperatures.

After analyzing the DSC scan for the sample with a milling time of 8 h (Figure 5b), the T_p1_ peak occurred at a temperature of 250.5 °C. The transformation was weakly exothermic.

**Figure 6:**
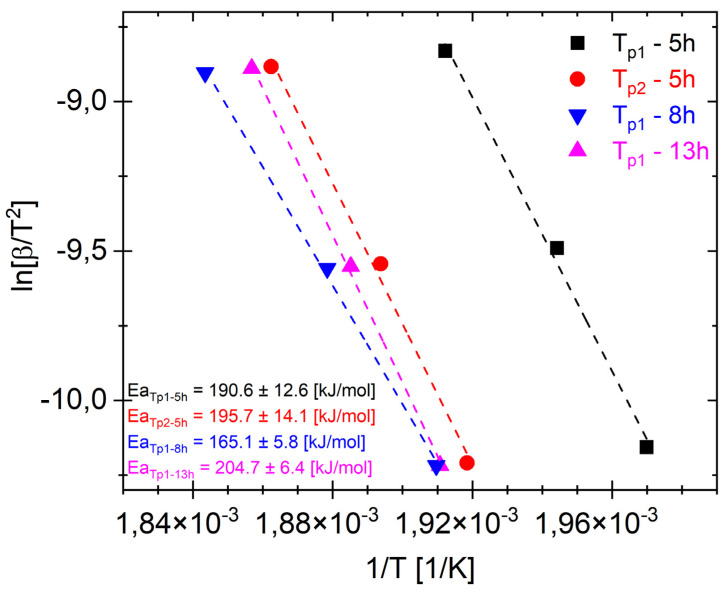
The results of the analysis of Kissinger activation energy Ea plot determined for first T_p1_ and second T_p2_ exothermal reaction for the Mg_65_Zn_30_Ca_4_Pr_1_ alloy powder milled for 5, 8, and 13 h.

Figure 6 shows Ea_Tp1_ and Ea_Tp2_ for different representative milling times. It should be noted that Ea_Tp2_ for 5 h and Ea_Tp1_ for 8 and 13 h probably correspond to the transformation of the same phase (MgZn_2_). The plots (Figure 6) show that the activation energies for phase formation Ea_Tp1_ after milling times of 5, 8, and 13 h are 190.6 kJ/mol, 165.1 kJ/mol, and 204.7 kJ/mol, respectively. And Ea_Tp2_ after 5 h is 195.7 kJ/mol. The low value of Ea_T1_ = 165.1 ± 5.8 kJ/mol for the alloy after a milling time of 8 h (Figure 6) may have been caused by two-phase transformation processes overlapping, and when the first transformation took place (Figure 4b and Figure 5b), the next phase began (T_p2_). Moreover, the XRD pattern shows that there is both an amorphous phase and the MgZn_2_.

It should be noted that EaT_p2_ for 5 h and EaT_p1_ for 8 and 13 h most likely correspond to the same phases. The obtained results (Figure 6) correspond to the activation energy value Ea = 207 kJ/mol of the MgZn_2_ phase obtained in [48].

**Figure 7:**
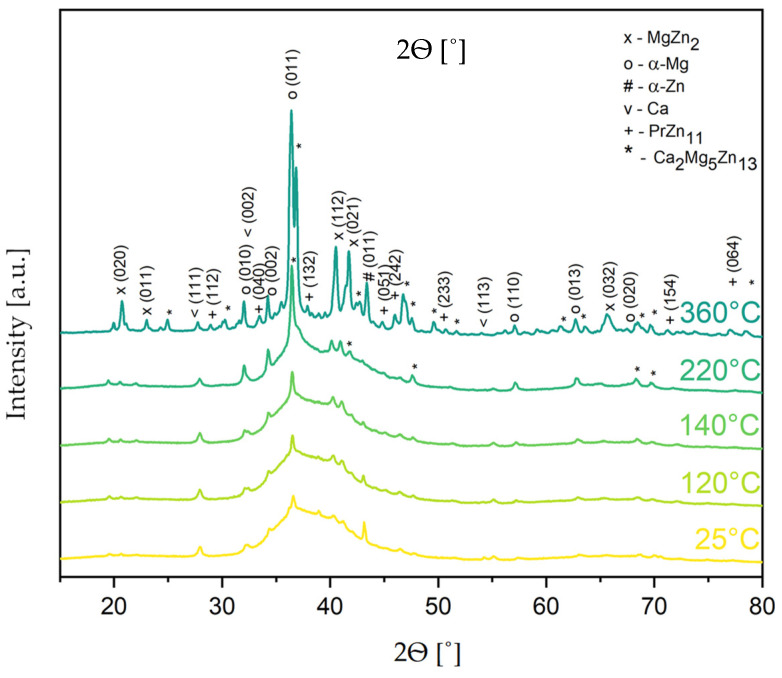
XRD patterns of the Mg_65_Zn_30_Ca_4_Pr_1_ alloy powder were milled for 20 h and heated at different temperatures (25, 120, 140, 220, and 360 °C). The powder heated from 25 to 140 °C, below the first exothermic reaction, shows a broadening of the maximum typical for amorphous phases and peaks for crystalline phases. The phases identified in the analysis were α-Mg (P6_3_/mmc) (98-005-2260), MgZn_2_ (P6_3_/mmc) (98-004-6006), α-Zn (P6_3_/mmc) (98-005-2259), and Ca (Fm-3m) (98-042-6932). After heating at 220 °C, some additional peaks (2θ = 36.8, 42.0, and 47.3) were observed, which were identified as the tetragonal Ca_2_Mg_5_Zn_13_ (00-012-0569) phase.

This claim is further supported by the XRD results featured in Figure 7.

The DSC method allows for tracing the change phase but does not identify the phase qualitatively. This is possible with a supporting characterization method, such as X-ray diffraction. To understand the origin of the exo- and endothermal heat effects observed in the DSC analysis (Figure 4a,b), the Mg_65_Zn_30_Ca_4_Pr_1_ powder alloy milled for 20 h was studied by using an X-ray diffractometer equipped with an in situ heating system. The XRD patterns of the Mg_65_Zn_30_Ca_4_Pr_1_ alloy powder for 25, 120, 140, 220, and 360 °C selected from the 25–360 °C temperature range are analyzed in Figure 7.

With the temperature increasing from 240 to 360 °C, the appearance of new peaks (2θ = 68.2 and 69.5) confirmed the presence of the Ca_2_Mg_5_Zn_13_ phase. The composition and homogeneity range of the Ca_2_Mg_5_Zn_13_ ternary phase in the Ca–Mg–Zn system were determined by Zhang [49]. This phase has a hexagonal structure with a P6_3_/mmc (194) space group and Sm_3_Mg_13_Zn_30_ prototype. Its presence was observed in the structure of the ZWO9203 and ZWX9203 alloy [50]. On the other hand, Wang [51] reported that the occurrence of the Ca_2_Mg_6_Zn_3_ phase influenced a reduction in the corrosion resistance of theMg_67_Zn_28_Ca_5_ cast magnesium alloy in simulated body fluid. In the literature, the presence of these phases was not found after sintering the Mg_65_Zn_30_Ca_4_Pr_1_ alloy at 350 °C [28].

In the powder that underwent heating at 360 °C, some additional peaks (2θ = 28.6, 33.0, 37.4, 44.5, 71.1, and 77.4) were observed, which are probably due to the forming of the PrZn_11_ (98-015-0495) phase (Figure 7).

The values of temperature determined by the DSC method were used for a determination of the temperature range for the sintering processes of the Mg-based alloy. These results indicate that the Mg_65_Zn_30_Ca_4_Pr_1_ alloy powder prepared by MA for different milling times (5, 8, 13, and 20 h) could be sintered at temperatures below 360 °C to generate a nanostructured multiphase alloy exhibiting a mixture of the amorphous phase and α-Mg (P6_3_/mmc), MgZn_2_ (P6_3_/mmc), α-Zn (P6_3_/mmc), and Ca_2_Mg_5_Zn_13_ phases. Sintering above these temperatures leads to the creation of the PrZn_11_ phase.

**Figure 8:**
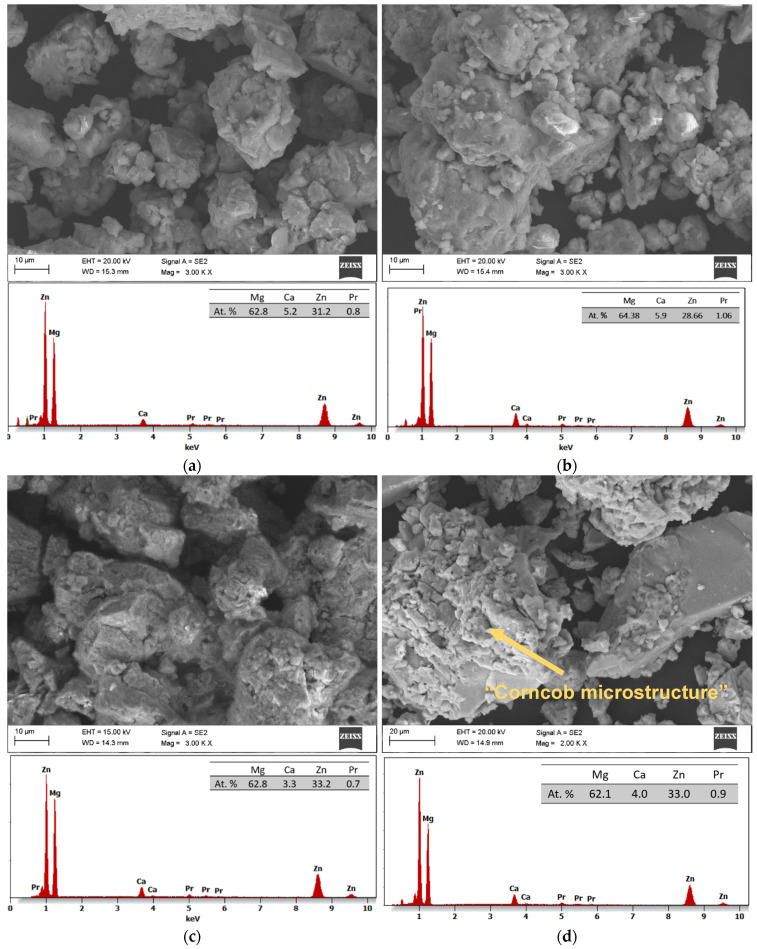
SEM images and EDS analysis of the Mg_65_Zn_30_Ca_4_Pr_1_ alloy powder prepared by MA for milling times of 5 h (**a**), 8 h (**b**), 13 h (**c**) and 20 h (**d**).

SEM images with EDS analysis of the Mg_65_Zn_30_Ca_4_Pr_1_ alloy powder prepared by MA for milling times of 5, 8, 13, and 20 h are presented in Figure 8a–d, respectively. The changes in the morphology of the powder during the MA process at different times are shown. The use of dissimilar powders in the mechanical alloying process is important in the amorphization process. The particle morphology of the alloy after 13 h of milling (Figure 8c) was different from that observed after 5, 8, and 20 h (Figure 8a,b,d, respectively) milling time. In Figure 8c, small particles are embedded into bigger ones that are visible. Particle morphology, including size and shape, is an important factor that influences the physical properties of the Mg_65_Zn_30_Ca_4_Pr_1_ alloy.

**Figure 9:**
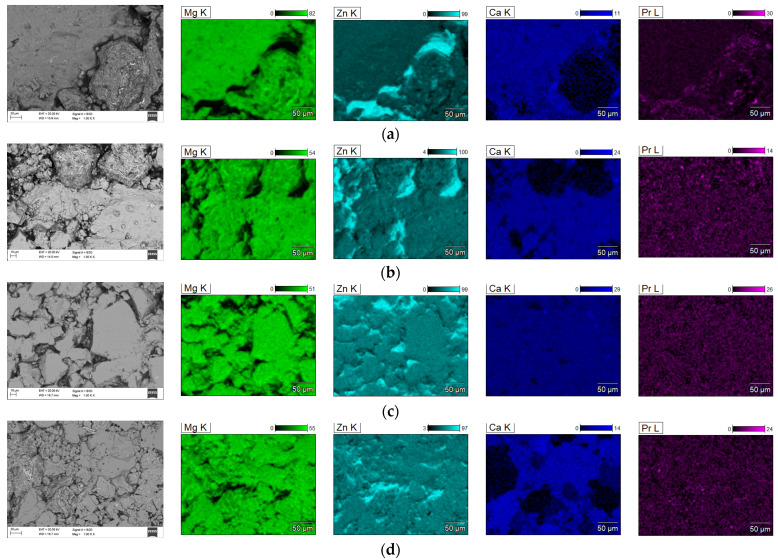
Maps of Mg_65_Zn_30_Ca_4_Pr_1_ alloy element distribution after (**a**) 5, (**b**) 8, (**c**) 13, and (**d**) 20 h of MA.

Figure 9 shows the element distribution maps in the produced alloy. The element distribution maps in the tested alloys indicate their high homogeneity and uniform distribution.

**Figure 10:**
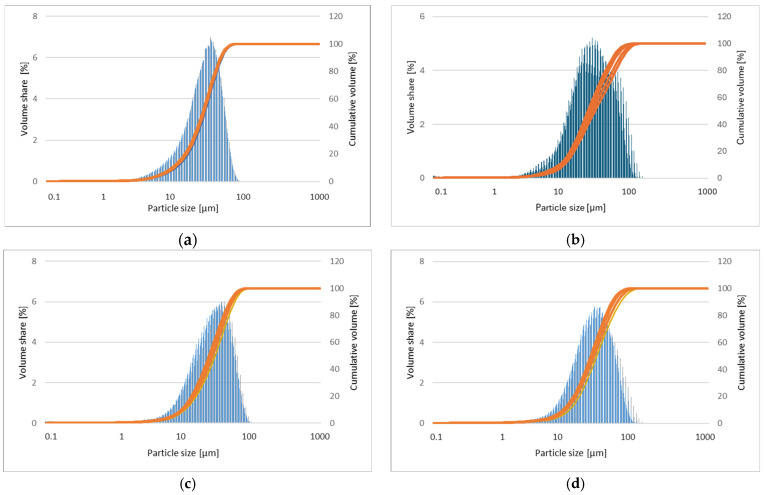
Particle size distribution results based on laser diffraction measurements for the Mg_65_Zn_30_Ca_4_Pr_1_ alloy produced by the MA method for milling times of 5 h (**a**), 8 h (**b**), 13 h (**c**), and 20 h (**d**).

The corresponding particle size distributions of powder mixtures milled for 5, 8, 13, and 20 h are presented in Figure 10a–d, respectively. The results of the measurement of the particle sizes of samples are presented in Table 1 [29].

In the first milling stages (Figure 8a,b), the particles tend to weld together and form large particles, leading to a wide range of average particle sizes. Longer milling times (Figure 8c,d) lead to particle crushing and work hardening via mechanisms such as fatigue fracture and brittle shell breaking. As the milling time increases, the particle size decreases, and the powder morphology assumes a more spherical shape. At this stage, the fracture tendency overcomes cold welding. When milling systems contain brittle–brittle materials, the harder, more brittle compound is embedded in the less brittle one (e.g., Zn is embedded within Mg). In Mg-Zn alloys, alloying is difficult because both Mg and Zn are brittle metals. The fine grains in brittle–brittle systems require a greater diffusion distance (Fick’low), making it difficult to alloy them at low temperatures. Ductile–ductile materials are easier to alloy due to their layered-type structure. Therefore, soft metals like Ca and Pr are introduced. The alloying mechanism of brittle materials differs from that of ductile–ductile materials [23,26]. Cracking occurs in hard, brittle materials, with minimal particle deformation and agglomeration due to welding. Deformation, cold welding, and cracking occur to varying degrees in both hard and ductile materials. Material transfer in mechanically alloyed brittle components may occur because of high temperatures, surface deformation, microstrain, hydrostatic stresses, or frictional wear. In addition, the proportion of fine particles increases with increasing milling time. The particle size in mechanical alloying is determined by the relationship between cold welding and fracture during milling. As shown in Figure 8c,d at higher milling times, the fracture is more pronounced than in cold welding. In the SEM image (Figure 8d), the “corncob microstructure” mentioned by [23] is visible. The milling time is one of the most critical parameters in milling and is usually selected to achieve a stable condition between the fracture and cold welding. In fact, after a particular milling time and reaching an equilibrium between the welding speed and the fracture rate (leading to a decrease in the average size of the average particles), a steady state is achieved. Higher milling times lead to increased contamination and the formation of undesirable phases [23]. Five different areas were selected in the sample for analysis to ensure the representativeness. The obtained results of the EDS microanalysis of chemical composition examined by scanning electron microscope (SEM) show conformity with the assumed chemical composition of the studied alloy (Figure 8b–d). It was shown that the typical EDS spectrum contains the lines that can be assigned to Mg, Zn, Ca, and Pr elements. The EDS analysis demonstrated the presence of alloying elements: Mg = 63 ± 1, Zn = 32 ± 2, Ca = 5 ± 1, and Pr = 1 ± 0.2 at. %, without other elements in the tested alloy. This indicates that the mixed powders are uncontaminated during the ball milling process. Element distribution maps (Figure 9) show alloy homogeneity and uniform element distribution. The results of the particle size distribution measurements presented in Figure 10 show a broad range of particle sizes. Similar, equiaxial particle shapes are observed for all powders (Figure 8 and Figure 10). A broad range of dimensions is observed for the sample milled for every measured time (Table 1). The Mg-Zn-Ca-Pr powder particles are characterized by a single particle size mode, with a typical particle diameter: D10 in the range of 9–13 μm and D90 in the range of 55–76 μm. The average particle sizes (D50) are 29.0, 30.0, 35.0, and 28.0 μm for the milling times of 5, 8, 13, and 20 h, respectively.

## 4. Conclusions

The main results of the research may be summarized as follows:

- It is feasible to synthesize Mg_65_Zn_30_Ca_4_Pr_1_ alloys by mechanical alloying (MA) with varying milling times in the range of 5–20 h;
- The structure of alloy powder is multiphase and consists of α-Mg, α-Zn solid solutions, a MgZn_2_ phase, and traces of unreacted Ca;
- After heating in a temperature range from 220 to 360°C, the Ca_2_Mg_5_Zn_13_ phase also occurs;
- The morphology of the milled powders reveals that the particles vary along the milling time. During the milling process, the particles are subjected to repeated welding and fracturing. With the increasing milling time, strong plastic deformations occur, and particle size decreases. The fluctuations in the particle size are characteristic of the MA process, as they are repeatedly subjected to cold welding, fracturing, and milling. This cyclic process leads to homogeneity in chemical composition;
- The results of the particle size distribution revealed similar, equiaxial particle shapes. The Mg-Zn-Ca-Pr powder particles are characterized by a single particle size mode. For the alloy after each milling time, there is a broad range of dimensions of particle size;
- MA is an effective method for the preparation of Mg-Zn-Ca-Pr powders, and in the next stage, their appropriate forming and sintering will enable the production of magnesium-based alloys with the structure, biodegradability, and mechanical properties to enable their use in medicine as potential materials for orthopedic implants.

## Figures and Tables

**Table 1:** The average particle size of Mg-Zn-Ca-Pr alloy powder milled at different milling times, where D10: the portion of particles with diameters smaller than this value is 10%; D50: the portion of particles with diameters smaller and larger than this value are 50%, also known as the median diameter; D90: the portion of particles with diameters below this value is 90%.

Milling Time [h]	Average Particle Size [µm]		
	D10	D50	D90
**5**	10	29	55
**8**	9	30	76
**13**	13	35	74
**20**	10	28	61

## Data Availability

The original contributions presented in the study are included in the article, further inquiries can be directed to the corresponding authors.
